# Bio-nano interactions: binding proteins, polysaccharides, lipids and nucleic acids onto magnetic nanoparticles

**DOI:** 10.1186/s40824-021-00212-y

**Published:** 2021-04-21

**Authors:** Lucía Abarca-Cabrera, Paula Fraga-García, Sonja Berensmeier

**Affiliations:** Bioseparation Engineering Group, Department of Mechanical Engineering, Technical University of Munich, 85748 Garching bei München, Germany

**Keywords:** Biomolecules, Adsorption, Iron oxide nanoparticles, Biocorona, Bioseparation, Bionanotechnology, Bio-nano interface, Interaction mechanism

## Abstract

The major interest in nanoparticles as an application platform for biotechnology arises from their high surface-to-volume ratio. Iron oxide nanoparticles (IONPs) are particularly appealing due to their superparamagnetic behavior, which enables bioseparation using external magnetic fields. In order to design advanced biomaterials, improve binding capacities and develop innovative processing solutions, a thorough understanding of the factors governing organic-inorganic binding in solution is critical but has not yet been achieved, given the wide variety of chemical and physical influences. This paper offers a critical review of experimental studies of the interactions between low cost IONPs (bare iron oxides, silica-coated or easily-functionalized surfaces) and the main groups of biomolecules: proteins, lipids, nucleic acids and carbohydrates. Special attention is devoted to the driving forces and interdependencies responsible of interactions at the solid-liquid interface, to the unique structural characteristics of each biomolecular class, and to environmental conditions influencing adsorption. Furthermore, studies focusing on mixtures, which are still rare, but absolutely necessary to understand the biocorona, are also included. This review concludes with a discussion of future work needed to fill the gaps in knowledge of bio-nano interactions, seeking to improve nanoparticles’ targeting capabilities in complex systems, and to open the door for multipurpose recognition and bioseparation processes.

## Background

Downstream processing (DSP) continues to be a challenge in biotechnology mainly due to two factors: the large amount of time needed to recover and purify targets, together with high processing costs [1, 2]. Depending on the target purity in the feed, DSP might require several steps to isolate particular molecules in the presence of a variety of contaminants from a fermentation broth which not only contains many different biomolecules, but also cell debris and salts [3]. Therefore, DSP often generates between 50 to 90% of the total production costs in most biotechnological products [4].

Iron oxide magnetic nanoparticles (IONPs) have mainly been gaining attention in the last two decades as they are inexpensive adsorbent materials [5] which allow harvesting whole cell systems [6, 7], and retention of different biological molecules, including proteins [8–13], nucleic acids [14–19], lipids [20, 21] and polysaccharides [22, 23]. While the global nanomedical market is expected to reach close to US$300 billion by 2022 [24], the global market for magnetic particle fluids could attain US$5.5 billion by 2024 [25]. Both markets are growing by ⁓15% annually, and they are closely related to magnetic nanoparticles. Therefore, the markets can be seen as an indicator of the economic potential behind IONPs. Additionally, for magnetite nanoparticles, a global market is expected to be valued at US $87.7 million by 2025 [26], growing ⁓10% annually. Practical and FDA approved products from IONPs in the market include: Feraheme™ for iron therapy; Nanotherm® (MagForce) for cancer treatment; Feridex® (USA) and Endorem® (Europe) as a magnetic resonance imaging (MRI) contrast agent for the liver; and GastroMARK™ as a contrast agent for the upper regions of the gastrointestinal tract [27]. Although the main approach is in biomedical applications, this material holds great promise for many other fields [28–32], and the forecasts above illustrate this potential.

The foremost advantages of IONPs are (1) their superparamagnetic behavior that enables easy handling and manipulation using an external magnetic field [33], (2) their low-cost synthesis (mainly iron salts in an alkaline environment and the chemicals for surface modification) [34] and (3) a high surface-to-volume ratio [35]. To adjust the surface properties, IONPs are coated or functionalized to avoid aggregation or biodegradation, and to enhance their selectivity for adsorption of target molecules [36–38]. A variety of reviews summarize different processes to prepare IONPs, from synthesis and characterization to functionalization [39–41].

The interactions between magnetic nanoparticles and biomolecules have been studied extensively, primarily in therapeutic applications [42], drug delivery [43–45], contrast image agents [46], antibacterial agents [47, 48] and bioseparations [49]. Nanoparticles in a biological medium are well known to be covered by biomolecules; these biomolecules form a ‘corona’ on the surface which alters the physico-chemical properties of the nanoparticles and confers on them biological attributes [50]. The so-called bio-nano interface is, therefore, created where classical forces, such as electrostatic, van der Waals, covalent, hydrophobic and steric interactions take place between a biological entity, the medium and a nanoparticle surface [51]. Numerous researchers have investigated the protein corona [52–55] due to its relevance in therapeutic applications of nanoparticles. Nevertheless, few studies focus on the other three major classes of biomolecules (lipids, polysaccharides and nucleic acids) [56–59] or on small molecules [60, 61], which are also highly relevant for the biotechnological industry. Hence, due to the low number of published studies, the mechanism of adsorption for other biomolecules has not yet been investigated systematically and thoroughly enough. Furthermore, an in-depth understanding of the adsorption of different biomolecular classes in mixtures, where they have to compete for the nanoparticle surface, is urgently needed to design better separation materials and enhance the efficiency of downstream processing.

In this review, we concentrate on superparamagnetic iron oxide nanoparticles ranging from a few to some hundreds of nanometers. This range is close to the size of most biomacromolecules, which are usually a few nanometers.

Although several reviews are devoted to interactions with nanoparticles [51, 62–68], none take a global view of the four main biomacromolecules and their derivatives to generalize about the behavior patterns, nor do they offer an overview of the state of knowledge in the field. We provide here, briefly, an overview of the fundamentals of the biomolecules adsorption on IONPs to set the stage for a detailed presentation of binding studies of the four main biomacromolecular classes focusing on spontaneous interactions with the nanoparticulate surface. Additionally, an emphasis is placed on studies with mixtures, where combinations of these molecules interact with the nanoparticles and compete for binding sites, opening exciting insights for future applications in bioseparation. Finally, we draw attention to the importance of further corona studies including all the molecules shaping it.

### Frontiers constructing the bio-nano interface

**Fig. 1:**
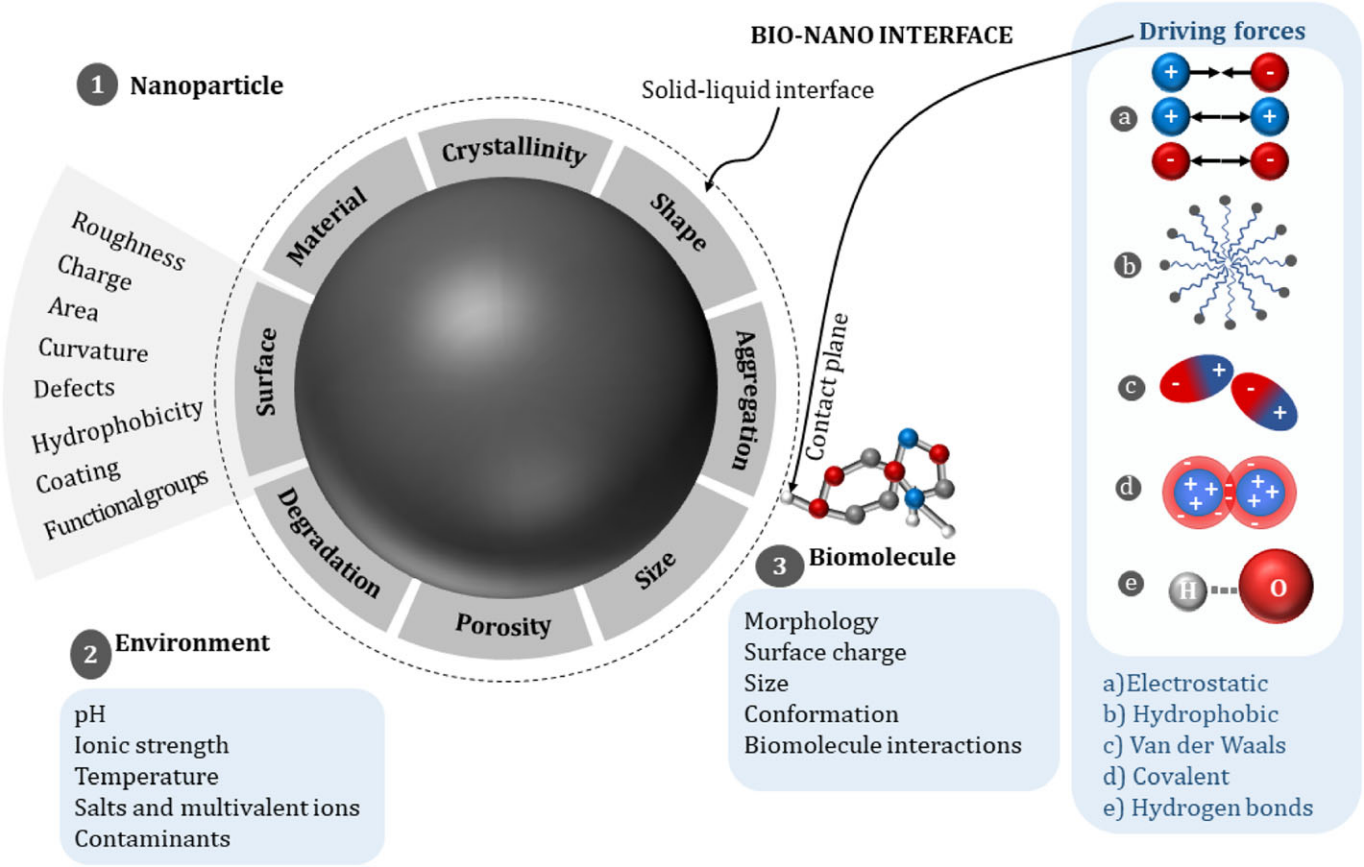
Frontiers constructing the bio-nano interface, which is constituted by the nanoparticle surface, the biomolecule and the medium. The characteristics of each element reshape the mechanism of interaction

In order to sketch the bio-nano interface, three important frontiers are often considered: (1) the nanoparticle surface which is characterized by its physico-chemical properties, (2) the biological material which converges with the solid-liquid interface at the contact plane, and (3) the surrounding milieu that, along with the particle, forms the solid-liquid interface (see Fig. 1) [51, 62].

The first element shaping the bio-nano interface is the nanoparticle itself. The identity of this component is obtained from its intrinsic attributes (illustrated schematically in Fig. 1); thus, an exhaustive characterization and understanding of the nanoparticle properties is crucial for studies of bio-nano interactions. The surface of IONPs is commonly modified with coatings and functional groups to adapt its charge, hydrophobicity and aggregation to better adjust its properties for the intended use. The isoelectric point (IEP) of bare IONPs has been reported to appear in a pH range mainly between 6.3 and 7.0 [69–72] Different proton affinities of the reactive surface groups lead to the existence of a surface charge both above and below the IEP: at basic pH, the nanoparticles acquire a total negative surface charge, and for an acidic pH a total positive surface charge. These proton affinities can be calculated using theoretical models, e.g. the CD-MUSIC model [73, 74]. Furthermore, protocols for the determination of the surface charge density have been published [75]. The presence of charged groups may be responsible not only for the stability and the agglomeration of the nanoparticles in suspension, but also for the interaction strength particularly when electrostatic interactions play the primary role. Accordingly, highly negative charged molecules, such as DNA, can be repulsed at high pH [17].

The second element at the bio-nano interface is the target biomolecule, which has individual characteristics, an assortment of shapes, charges, sizes and conformations in complex mixtures. These features dictate the particular functional groups exposed to the surrounding media and define the interaction with organic and inorganic materials. The third element of the interface is the medium. Its importance lies in its ability to alter the inherent characteristics of nanoparticles and biomolecules, such as surface charge (zeta potential), stability (biodegradability), hydration, valence state and electron transfer capability [51]. The medium also defines the ions which can compete for a site at the surface, decreasing the free adsorption sites for the molecules of interest and directly influencing the affinity and activity of the target molecules for the surface. The complex interplay of the chemical and physical parameters arising from these three elements dictate the adsorption process, which can be understood as a solid-liquid extraction process with partitioning coefficients for the different components in the mixed system.

Physico-chemical interactions take place between the nanoparticle surface and the biological compounds at the bio-nano interface [51]. At first, the forces collaborating in the interaction between the biomolecules and the nanoparticles look to be similar to those in classical colloidal systems [76]. The interactions can generally be divided into physisorption and chemisorption [62]. While physisorption relies on the attraction the adsorbing elements have for the surface while chemically unchanged (e.g. van der Waals, electrostatic and so-called hydrodynamic interactions), chemisorption appears when electron sharing occurs at the binding site typically developing stronger links (e.g. covalent bonds or hydrogen bonds) [62]. Moreover, these interactions may appear between the nanoparticle and the biomolecule, as well as between biomolecule-biomolecule or nanoparticle-nanoparticle, generating large scale changes, such as aggregation or dissolution [51]. In this review, these interactions, taken from different publications, are presented to evaluate their importance for binding patterns between biological substances and nanomaterials.

## IONPs as adsorbents of biomolecules

In the tireless quest to understand the mechanisms at the nano-bio interface, much research has been carried out on the adsorption of individual molecules assuming a steady state. However, the solid-liquid interface is a dynamic system, and therefore transient coronas are formed over time. In the following subsections, we examine the current knowledge of the four main biomolecules and their building blocks in interaction with iron oxide nanoparticles as scaffolds. The section concludes with a table containing some examples from the literature of different biomolecules in interaction with IONPs.

**Fig. 2:**
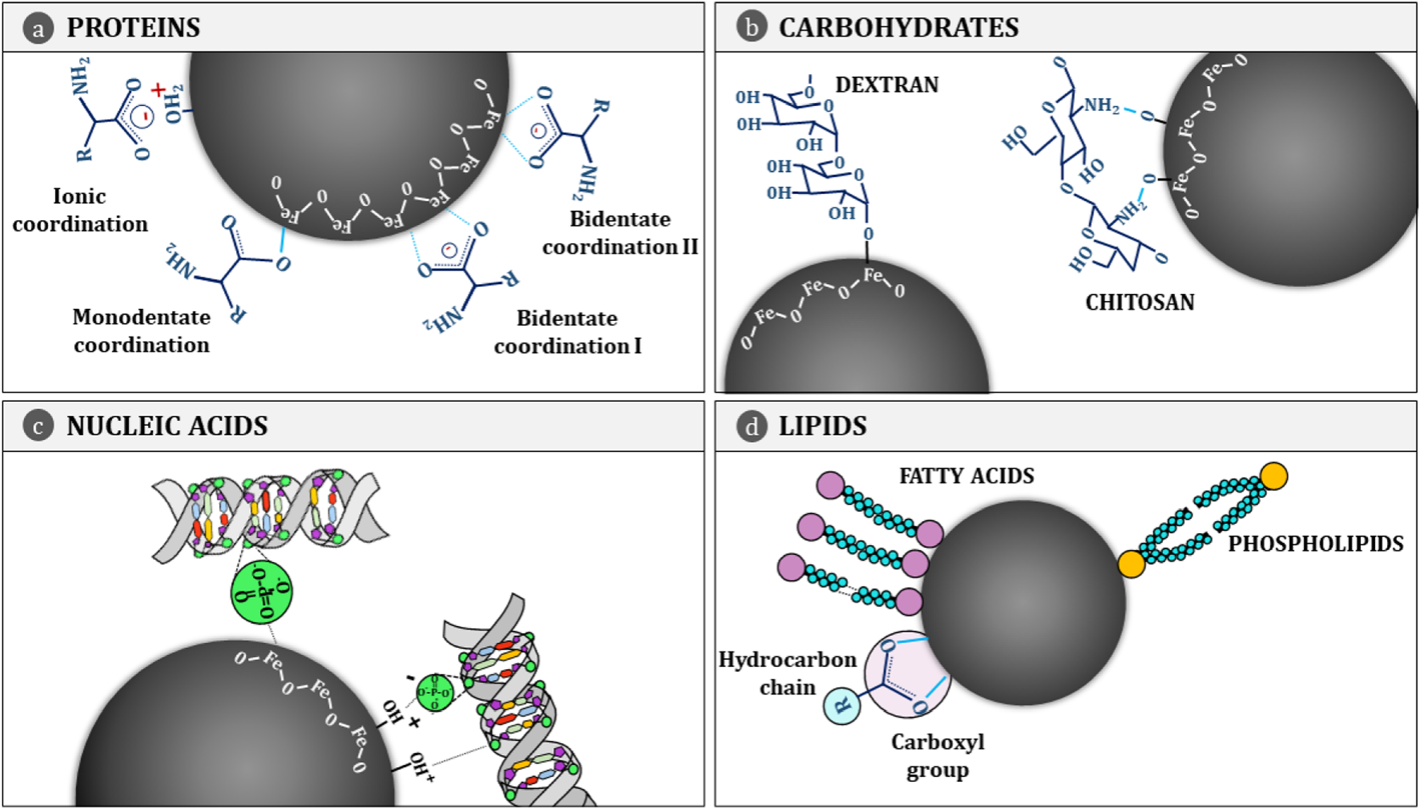
Manifold adsorption mechanisms. **a** Amino acids and peptides, building blocks forming proteins, are bound to the IONPs surface mainly by the carboxylic group using three different mechanisms: ionic, monodentate and bidentate coordination [61, 77, 78]. **b** Carbohydrates attach to the surface via the hydroxyl, amino and carboxylic groups [79]. **c** DNA interacts with IONPs through the phosphate group [15, 49]. **d** Fatty acids use the carboxylic group to join to the nanoparticle [80], while phospholipids use the phosphate group (head) or the hydrophobic chain (tail) depending on the hydrophobicity of the surface [81]. Both form bilayers around the surface

In Fig. 2, we offer an overview of the adsorption mechanisms involved in the interactions of amino acids, lipids, nucleic acids and carbohydrates with the surface of magnetic nanoparticles.

### Proteins

Proteins are large biomacromolecules constructed of peptides, chains of building blocks called amino acids covalently joined [82]. The function of the proteins depends on the structure, and hence the native stage must be preserved in order to have functional molecules [83].

Proteins are the most studied biomacromolecular group in interaction with magnetic nanoparticles. Some recent reviews offer an overview of characteristics related to the interaction of proteins and IONPs for bioseparation and other application fields [5, 49, 65–68, 84–88]. These molecules are known to easily bind directly after incubation with surfaces and immediately cover them via electrostatic interactions, hydrogen bonds, hydrophobic interactions and coordinative bonds [89]. However, a complex interplay of factors dictates the extent and specificity of the binding.

The first factor to analyze in the interaction are the characteristics of the nanoparticle. Upon the composition of the nanoparticle (material, shape and size), which manifestly affects the protein binding, the surface chemistry such as charge and hydrophobicity has a higher impact [90]. However, these physico-chemical properties of the nanoparticulate system, for instance the size and the surface charge, change after binding [52]. The surface charge influences the adsorption of certain proteins [91]. Anionic nanoparticles strengthen the adsorption of proteins with an isoelectric point (IEP) greater than 5.5, i.e. IgG, while cationic nanoparticles intensify the adsorption of proteins with an IEP below 5.5, i.e. albumin [92].

The second factor to analyze is the environment [93]. In biotechnological applications, proteins are generally located in highly complex buffered mixtures [94]. The ions in the solution buffer presumably compete with biomolecules for the adsorption sites. This has been verified by Blank-Shim et al. (2017) by using amino acid homo-hexamers that had been immobilised on a cellulose membrane and had been contacted with bare IONPs at different pHs using three different charged species (tris, phosphate and citrate) [95]. The authors noted that in phosphate buffer saline (PBS), the binding of positively charged peptides increases. They described this interaction based on electrostatic attraction as the nanoparticle surface becomes more negatively charged. On the other hand, when using a Tris buffer, the adsorption is lower than in PBS. Tris molecules have a positive charge at those pH values, and therefore the ions compete with the positive peptides for the negative surface.

The third factor influencing the interaction is the construction of the protein. The characteristics of proteins are mainly defined by the amino acid succession that determine the size, charge and shape of the protein. Regarding the tridimensional structure, proteins can experience structural changes due to the interaction with IONPs, where the effect increases at higher relative concentrations of the nanomaterial. Mahmoudi et al. (2011) analyzed CD spectra of transferrin glycoproteins, which contain two iron binding sites [8]. The authors found that the exposure of transferrin to bare and PVA-coated IONPs in PBS at pH 7.4 results in conformational changes of the protein from a compact to an open jaw structure, showing irreversible changes [8]. Smooth iron release and, hence, a reversible process, can be obtained at pH 5.5, due to the fact that transferrin in its natural environment spontaneously releases iron at this pH [96]. One point to further consider is the molecular relaxation or protein spreading, which leads to changes in the surface distribution over time. This phenomenon occurs due to environmental conditions or through strong interaction forces, modifying the orientational arrangement of adsorbed proteins that could lead to activity inhibition (for example, inhibition of enzyme activity) [97]. This effect should also be evaluated in competitive, multiprotein mixtures in which residence time, concentration ratios, and spatially dependent adsorption maxima are taken into consideration.

Generally, studies of amino acids can help to predict a pre-determined behavior in complex and larger structures like proteins [95]. However, amino acid interactions do not help to consider large molecule effects such as steric effects [78], conformation and organization of the protein on the surface. We consider them in this review to help understand protein-IONPs interactions and because amino acid are used in most of the mechanistic descriptions.

Although amino acids are known to form a chemical bond of the chelate type through the iron ions on the surface and the carboxylate group of amino acids [60], the mechanisms in nanoparticles are still being researched as the results in the literature are contradictory. The binding mechanism of l-lysine on IONPs has revealed a unidentate coordination between the carboxylic group and the metal ions, resulting in chemisorbed interactions [98], leaving the amino groups exposed to the medium which forms small molecular associates in acidic conditions (i.e. pH 2.0) [60]. Glutamic acid uses the alpha or side chain carboxyl groups and both carboxyl groups simultaneously. Histidine binds with its imidazole ring while serine via the hydroxyl group [61]. Regarding L-aspartic, Mikhaylova et al. (2004) report that the interaction with IONPs is through the second carboxylic group and not the amino group or the α-carboxylic group [99]. On the other hand, Puŝnik et al. (2016) attribute different adsorption mechanisms depending on the concentration of Asp [60]. Using acidic pH where the carboxyl groups are protonated at low amounts of this amino acid, the adsorption takes a monolayer form mediated by both carboxyl groups, while at high concentrations, the formation of molecular associations is carried out with the α-carboxylic group on the surface and the side carboxyl group oriented outward. Cysteine exhibits the highest adsorption capacity compared with six different amino acids. It interacts via the carboxyl group and side chains in magnetite nanoparticles, which are bound by electrostatic interactions in a multilayer manner [61]. However, strong non-covalent interactions reveal similar bonding strength of cysteine onto gold particles when compared to the carboxylate groups of aspartic acid and the phenyl ring of tyrosine [64].

Peptides are small amino acid sequences whose distribution over the surface can adopt multiple spatial arrangements. Slocik et al. (2010), for instance, proposed two conformations that could take place at the same time for peptides: with a cysteine in the N-terminal or in a flat-on configuration with multi-dentate bindings [64]. However, these arrangements are frequently not differentiated due to measurement limitations and thus, adsorption is described as an average number of peptides on the surface [64].

Another important factor to consider when analyzing the interaction of proteins with nanoparticles are the driving forces at the bio-nano interface, which play an important role in the interfacial ‘battleground’. Regarding electrostatic interactions, considerable uncertainty remains about whether or not they play the main role in the adsorption of proteins in aqueous systems. Some studies report a direct relationship between adsorption and other factors such as serine phosphorylation [100]. Moreover, positive peptides are allowed to bind to positively charged surfaces, though this bonding only seems to occur when the surface is slightly charged (e.g. + 3.7 mV [95]). Nevertheless, at high net charges, only the oppositely charged peptides are joined. Presumably, magnetic nanoparticles present heterogeneous charges on the surface when close to the IEP, and thus both kinds of charged molecules (positive and negative) can weakly bind [101].

The adsorption of individual molecules is usually described using thermodynamic models, mainly the Langmuir and Freundlich isotherms. For proteins and their building blocks, very different loading capacities are reported in the literature. Some examples for bare or easily functionalized iron oxide nanoparticles range from ~ 10 to 490 mg g^− 1^ for amino acids [60, 61, 102], from 20 to 280 mg g^− 1^ for peptides [77] and from 25 to 570 mg g^− 1^ for proteins [5, 11, 103–109].

### Carbohydrates

Saccharides participate in a variety of biological activities, mainly to provide structure and energy storage sources. Polysaccharides are formed by monosaccharides linked by glycosidic bonds in the form of linear or branched structures [79].

Many saccharide structures change only in the orientation of hydroxyl groups [110]. However, they can also undergo changes on length, charge, monosaccharide sequence and stereochemistry [111], making it challenging to properly identify patterns in their adsorption on nanoparticles, as each polymer possesses unique physico-chemical properties. Intricate analytical methods are required to differentiate saccharide molecules in aqueous solutions, a fact which could explain why few interaction studies have focused on this macromolecular class.

Polysaccharides are often employed as colloidal stabilizers of magnetic nanoparticles during synthesis and for surface modification [112–114], as they are biocompatible, non-toxic, renewable and contain functional groups that allow further functionalization [79]. The most common polysaccharides employed for surface modifications are agarose, alginate, chitosan, dextran, hyaluronic acid, heparin, pullulan, starch and carrageenan [115]. The coating yields new properties at the surface and prevents the formation of large aggregates [35].

Several coating methods exist, in-situ or post-synthesis [79]. Chemical co-precipitation is the most common in-situ method to coat magnetic nanoparticles with polysaccharides. Here Fe^2+^ and Fe^3+^ ions are mixed together with a polysaccharide followed by the addition of ammonium or sodium hydroxide. The polysaccharides are forced to bind to the surface through chemical interactions or cross linking. Post-synthesis methods include encapsulation, covalent binding and adsorption.

Regarding negatively charged polysaccharides, alginate is an anionic polysaccharide enriched by carboxyl groups which contains β (1,4) linked D-mannuronic acid and α (1,4) linked L-guluronic acid. The negatively charged COO- groups of the alginate polymer interact with the Fe cations establishing stable IONPs-alginate complexes forming a pre-gel state [37, 116]. Heparin is a highly negatively charged polysaccharide formed by 1,4-linked uronic acid or l-iduronic acid and d-glucosamine which can include sulfo groups. Naturally found on the surface of all eukaryotic cells [79], heparin has been adsorbed on magnetite nanoparticles dispersed in water at pH 5.0 to serve as a coating, obtaining IONPs with a negative charged surface [117]. Chitosan, a positively charged polysaccharide, is a hydrophilic, non-toxic and biodegradable polymer [37] with poor solubility; therefore, in aqueous solutions, this polysaccharide requires acetic acid to dissolve. The coating of iron oxide nanoparticles with chitosan is carried out during synthesis, possibly because little [112] or no spontaneous adsorption takes place. FT-IR measurements show strong hydrogen bonds formed between the oxygen of Fe_3_O_4_ and the hydrogen of amino groups but not through the hydroxyl group of chitosan [118]. The hydroxyl groups remain exposed, so the surface acquires a positive charge due to the smaller electronegativity of hydrogen compared to oxygen [118].

Little has been reported on a spontaneous adsorption of carbohydrates on IONPs. Hydrogen bonding is the primary adsorption mechanism of polysaccharides with a weak bond, but an interplay of interactions is commonly reported [22]. For instance, the high affinity of starch to iron oxide is initially due to the hydrogen bonding, but with time it leads to the formation of chemical complexes [22].

As described in the proteins section, one way for analyzing the interaction is to study the characteristics of the nanoparticle. As previously discussed, the crystalline structure affects the arrangement of Fe^2+^ and Fe^3+^ ions, hence the position of hydroxyl groups in aqueous medium [79]. Veloso et al. (2020) report that around 20% more starch is adsorbed on hematite than on magnetite [22].

Carbohydrate characteristics are another factor influencing the interaction with the IONPs. Different functional groups of polysaccharides can react with the solid surface, and these groups are mainly comprised of hydroxyl groups as well as carboxylic groups (alginate, hyaluronic acid and gum arabic) and amino groups (chitosan) [66]. Chain length also plays a role in adsorption. Starch is composed of D-glucose units joined by glycosidic bonds [79], while dextrin is produced by hydrolysis of starch and, consequently, contains a lower molecular weight. This length influences the number of hydroxyl groups available for the interaction, and hence dextrin shows a lower adsorption capacity [22].

In contrast to proteins, the adsorption isotherm of polysaccharides is usually fitted to the Freundlich model [22, 23]. In starch-based wastewater, IONPs were used to study the adsorption behavior of polysaccharides, comparing three adsorption isotherms (Langmuir, Freundlich and Sips) [23]. In acidic conditions, IONPs were in contact with diluted waste samples containing from 7000 to 8000 mg L^− 1^ of polysaccharides [23]. The Freundlich model proved to be the best to describe the process and indicated multilayer coverage on the surface with moderately favorable adsorption [23]. Similar results have been shown by Veloso et al. (2020), who studied the interactions of four natural polymers – corn starch, dextrin, humic acid and cellulose – with non-nanoscale hematite and magnetite materials [22]. The behavior of all these polysaccharides matched predictions of the Freundlich model.

The glycome also includes glycoconjugates, comprised of a carbohydrate chain called glycan and a lipid or protein molecule attached covalently [69]. Polysaccharides combined with proteins form glycoproteins or peptidoglycans, and with lipids polysaccharides form glycolipids [60]. These glycoconjugates, which can differ in glycan sequence, length and connections, are obtained through glycosylation, a post-translational modification made by eukaryotic systems that enables immune cell recognition [119]. Other post-translational modifications can be carried out, such as phosphorylation, acylation, ubiquitylation and so on, producing a great variety of biomolecules, therefore too many to be included in this review. However, as an illustration, antibodies, categorized as glycoproteins, are commonly conjugated with IONPs to identify and localize specific cells [120, 121]. New insights have to be gained on how these modifications affect the interaction of biomolecules with inorganic materials.

### Nucleic acids

Nucleic acids are polymers that encode the genetic information of a cell and guide protein synthesis. These polyanionic molecules are composed of nucleotides, which contain an anionic phosphate group, a pentose and a base (adenine, guanine, thymine, cytosine or uracil) in a single-stranded (RNA) and a double stranded form (DNA) [122].

The formation of hydrogen bonds and electrostatic interactions are responsible for the adsorption of DNA in IONPs. The interaction happens through the Fe-O-P bond due to the negative charge density distributed over the phosphate group located on the backbone of the molecule [15, 49]. In addition to the phosphate group, RNA/DNA can be adsorbed on surfaces via nucleobases, as has been described for metallic nanoparticles, such as gold nanoparticles or for carbon nanotubes [123]. Vanyorek et al. (2019) conclude that the principal mechanism of DNA binding is the formation of hydrogen bonds between the DNA phosphate group and the hydroxyl groups exposed on the nanoparticle surface [16]. Superparamagnetic iron-oxide nanoparticles were used to reversibly bind purified pBAD type vector DNA from *E. coli* in aqueous solution at pH 8.0. Although the binding capacity is not identified in the study, electrophoretic data demonstrated that no DNA remained in the supernatant, while DNA was found in the first and second elution, suggesting that IONPs adsorbed all the DNA used [16]. The authors suggest that the presence of hydroxyl groups on the nanoparticle surface yields a great dispersibility in the aqueous phase and facilitates the formation of hydrogen bonds with the DNA. This study shows that even though the IONP surface is characterized as negatively charged, the negative DNA molecule binds to it given the heterogenous charge of the nanoparticle surface, due to the presence of hydroxyl groups.

In contrast, a study using unmodified, coated and functionalized IONPs reveals that the interaction mechanism of oligodeoxynucleotides (ODN) and plasmid DNA (pDNA) is mediated by electrostatic interactions between positively charged surfaces at pH 4, where hydroxyl and amino groups are protonated (−OH_2_^+^;-NH_3_^+^), and the negatively charged phosphate backbone [17]. Metal oxide nanoparticles are more protonated at low pH and then, as expected at these conditions, DNA binds tightly to the surface [123]. The IONPs functionalized with TRIS produced the greatest amounts of bound ODN, probably attributable to a high surface area [17]. Although not mentioned, as zeta potential measurements are not included in their study, commercially used silica coated nanoparticles are well known to present negative charges in a wide range of pH [124]; however these nanoparticles obtained the largest amount of adsorbed negatively charged pDNA. At pH 4.0, the zeta potential seems to be close to a neutral charge (~ − 15 mV) and consequently electrostatic repulsions no longer play the primary role in the interaction between the polyanion DNA and the nanoparticles, being replaced by hydrogen bonds.

Dehydration plays a significant role in the adsorption of macromolecules in aqueous systems where it contributes to the development of hydrophobic interactions and hydrogen bonds [125]. In their native state, nucleic acids are surrounded by water molecules which make the DNA molecule soluble, mediated by the formation of hydrogen bonds with the oxygen from phosphate groups. Sterically accessible oxygen atoms on the nanoparticle surface, or silanol groups from silica coatings also support the binding of water molecules by hydrogen bonds. During dehydration, the water molecules are released, commonly by using chaotropic agents such as guanidinium chloride, SDS and urea, forming a hydrophobic environment. As a result, hydrophobic interactions are promoted. Furthermore, aqueous solutions with low pH values promote protonation of hydroxyl groups on negatively charged surfaces, developing direct bio-nano interactions by forming hydrogen bonds between the phosphate backbone and the surface. This phenomenon is often seen in DNA adsorbed at silica coated nanoparticles [17].

As previously found for peptides and proteins [77, 106], DNA can easily be desorbed using 0.5 mM of sodium phosphate (pH 7.6) which competes for the surface [123]. However, the particular DNA sequence can also play a minor role as a result of potentially weak base interactions or secondary structures. Comparing the effect of four types of 15-mer on IONPs at pH 7.6, all of the homopolymers were adsorbed, but less adsorption was conformed in FAM-G15 as a result of the formation of a secondary structure [18].

Iron oxide nanoparticles bind to nucleic acids (e.g. DNA), even though they are not functionalized [16], but aggregation is then observed. Aggregates of naked magnetic nanoparticles [16, 17] or APTES functionalized IONPs [17] decrease the DNA binding capacity as the available surface area is reduced [63].

Over a quarter of the total weight of DNA molecules is phosphate [126], which is located at the backbone. This enhances adsorption capacity as various phosphate groups bind to the nanoparticle [49]. Furthermore, this characteristic allows stronger interactions due to the higher number of nanoparticle binding sites used, preventing displacement by other molecules.

The DNA conformation is also important because it impacts the distribution and accessibility of phosphate groups in supercoiled DNA: phosphate groups localized within the structure cannot participate in the interaction, and hence a lower number of adsorbed molecules are obtained in comparison with linear structures such as oligodeoxynucleotides [17]. Additionally, the lower capacity obtained from 20 nm-IONPs (i.e. 105 nM DNA equivalent to 55 oligonucleotides of 15 nucleotides) in comparison with 20-nm gold nanoparticles (200 DNA molecules) leads to the assumption that DNA wraps the surface rather than forms an upright conformation as described for gold nanoparticles [18].

DNA can also bind by direct covalent binding to naked iron oxide nanoparticles. Plasmid (pDNA) containing GFP genetic code was chemisorbed onto naked surface active maghemite nanoparticles [19]. Nanobioconjugate formation takes place with the surface exposed iron (III), and the negatively charged phosphates from DNA are bound by bidentate coordination forming a chelate complex. Note that after the DNA has been immobilized, the surface is not completely saturated, and sites are still available for other biomolecules [19].

Unfortunately, the studies referred to in this section rarely characterize the nanoparticles after adsorption or show adsorption isotherms, which is necessary to understand the adsorption mechanism and to compare the capacities between materials and with other biomolecule types.

### Lipids

Lipids are hydrophobic organic molecules, insoluble or only partially soluble in water but soluble in non-polar solvents. These molecules comprise a broad variety of compounds, such as fatty acids, phospholipids, sterols and terpenes, among others [127].

Fatty acids are formed by a carboxylic acid and an aliphatic chain. Some examples of fatty acids such as oleic acid [80, 128] and ricinoleic acid [128, 129] have been widely reported for coating IONPs to improve the colloidal stability of the nanoparticles.

Carboxylate groups belong to the main factors in the binding of fatty acids with nanoparticles. The interaction of carboxylic functional groups has already been described in the ‘Proteins’ section for some peptides. Fatty acids have shown a bilayer formation around the IONPs surface, in which, the polar carboxylic head group binds to the surface in the inner layer, and thus the non-polar, hydrophobic tail is directed to the solvent. The second layer is formed by the non-polar tail groups that interact with the tail groups of the attached fatty acids, and the carboxylic acid heads of this second layer are exposed to the aqueous medium [80]. The bilayer conformation provides high hydrophilicity to the structure exposed to the aqueous surrounding, creating stable colloidal nanoparticles and preventing the aggregation of the colloids [130]. Studies with oleate suggest that the carboxylate groups are adsorbed to the surface by bidental and ionic coordination of the inner layer, forming dimers at high concentrations, and further multilayers at even higher concentrations. Additionally, the adsorption of the oleate is described as small, carpet-like, double layer islands that expand on the surface until it is completely covered [80]. Oleate acts as a non-ionic surfactant in which, at high concentrations where the critical micelle concentration (CMC) is exceeded, micelles are formed simultaneously and compete with the adsorption of the surfactant on the surface [80]. Nonetheless, other studies challenge this result. The interaction of oleate and iron oxide surfaces is stronger than the formation of micelles [130], and thus the decrease of ‘free’ surfactant in the fluid induced by the adsorption of the surfactant prevents reaching the CMC, i.e. the formation of micelles [131]. Cano et al. (2012) analyzed the interaction of fatty acids from organic solutions and vegetable oils using IONPs. Through FTIR, the carboxylic group has been shown to bind to the surface using covalent interactions in a bidentate and bridging form; these experiments yielded an oleic acid saturation adsorption capacity of 125 mg g^− 1^ [132]. Other researchers report about 300 mg g^− 1^ at room temperature [80].

Another class of lipids are the phospholipids, which are amphiphilic molecules formed of a polar phosphate group (head) and two hydrophobic carbon tails; these molecules interact with nanoparticles depending on the hydrophobicity of the surface [81]. Studies using self-assembly methods for coating with phospholipid-PEG first use surfactants, such as oleic acid, to stabilize the nanoparticle and make it hydrophobic [20, 133–135]. After the first coating, phospholipids i.e. DSPE or DMPE, interdigitate with the tails of oleic acid via hydrophobic interactions. It has also been observed that phospholipids, such as DOTAP, can self-assemble into naked IONPs, forming a monolayer. Importantly, IONPs create a hexagonal array where the monolayer interacts with the adjacent layer forming a bilayer, which is compressed due to the effect of van der Waals and magnetic dipolar forces [21]. Additionally, the surface curvature of IONPs influences the density of phospholipids on the surface. Lower curvatures, present in larger particles (> 20 nm), lead to a different assembly density of the phospholipids: tails form parallel alignment in stretching mode and heads stack leaving gaps given their spherical shape, reducing lipid density at the surface and augmenting the hydrophobicity of the nanoparticle. On the contrary, small nanoparticles (< 20 nm) lead to an increased lipid density and produce thinner shells due to the angle formed by the phospholipids [133, 136].

There are many other lipids, such as saccharolipids or sterols, but they are less often studied in their interaction with nanoparticles. One example is the use of IONPs for cholesterol separation [137–139]. Some other complex structures, such as magnetoliposomes, contain hydrophobic IONPs by entrapping them within a spherical phospholipid bilayer, converting these structures into magnetically controlled platforms for drug delivery systems [140]. These lipid-based molecules are not included in this study due to their different chemical makeups and the low number of studies examining them. However, they represent a solid opportunity to conduct in-depth research on bio-nano interactions in the future.

### Overview of interaction forms and gaps in knowledge

Several examples showing the forms of interaction of individual molecules which we have presented in the sections above can be found in Table 1. These examples should provide an overview of the interaction mechanisms discussed and not to present a comprehensive overview of all biomolecule - IONPs interaction studies, which would be far beyond the scope of this review.

**Table 1:** Mechanisms of interaction reported for biomolecules onto iron oxide nanoparticles

Target biomolecule	Nanoparticle type and size	Interaction mechanism	Ref.
**AMINO ACIDS, PEPTIDES AND PROTEINS**			
Transferrin	Bare/PVA coated IONPs (5–10 nm)	By affinity using iron binding sites	[8]
Lysine	IONPs (9.7 ± 1.5 nm and 14 nm)	Through the carboxylic group and molecular associates at high concentrations	[60, 61]
Aspartic acid	IONPs (9.7 ± 1.5 nm)	Both carboxyl groups attached to the surface, through the side carboxyl group and associates.	[60]
Glycine	IONPs (14 nm)	Via carboxyl group in ionic or bidentate coordination	[61]
Glutamic acid	IONPs (14 nm)	Bridging mechanism by the α- or the side chain carboxyl group	
Serine	IONPs (14 nm)	Formation of ionic or bidentate bridging complexes	
L-arginine, L-lysine L-glutamine and glycine	IONPs (NA)	Electrostatic interactions	[141]
Homo-peptides	IONPs (14 nm)	Electrostatic interactions	[95]
Plasma proteins	Azaleic acid coated IONPs (10 nm)	Hydrophobic interactions	[54]
Glu8 peptides	BION (5–20 nm)	Carboxylate groups	[77]
**FATTY ACIDS AND OTHER LIPIDS**			
Sodium oleate	IONPs (10 nm)	Bidentate coordination: carboxylate group of sodium oleate and hydroxyl groups of the IONP surface.	[80]
		Ionic interaction of carboxylic groups on the secondary layer	
Oleic acid, palmitic acid, stearic acid, and linoleic acid	IONPs (8 nm)	Covalent binding	[132]
Multilipids, including DSPE-mPEG and DSPE-PEG-PDP	Oleic acid coated IONPs	Hydrophobic interactions between the oleic acid tails and DSPEPEG tails (lipid stitching)	[133]
	(10–30 nm)		
DOTAP	IONPs	Hydrophobic, van der Waals and magnetic dipolar force forming a bilayer	[21]
	(11 ± 1.3 nm)		
**NUCLEIC ACIDS**			
DNA	IONPs (15.3 nm)	Hydrogen bonds	[16]
ODN	TRIS coated IONPs (2.6 nm)	Electrostatic interactions: hydroxylic groups and phosphate	[17]
pDNA	Silica coated IONPs (9.8 nm)	Electrostatic interactions: hydroxylic groups and phosphate	[17]
DNA	IONPs	Electrostatic interactions with phosphate	[123]
Salmon DNA	Silica coated IONPs (70 nm)	Electrostatic interactions	[142]
DNA	Amino functionalized silica coated IONPs (25 nm)	Electrostatic interactions	[143]
		(amino groups and phosphate backbone)	
**CARBOHYDRATES**			
Chitosan	IONPs (11 nm)	Electrostatic interactions	[112]
Alginate			
Polysaccharides	IONPs (8.41 ± 0.94 nm)	NA	[23]

The studies presented in the previous sections consistently show that all four varieties of biomacromolecules spontaneously interact with IONPs in aqueous suspensions. Despite the fact that many studies focus on proteins, they typically analyze model systems (including amino acids and peptides), i.e. systems with only one or a few molecules. Polysaccharides are also a popular research topic regarding their adsorption to IONPs. However, the investigations primarily focus on their use as a coating material, with only a few studies providing data on the spontaneous adsorption of these polycarbohydrates to nanoparticles. Together with nucleic acids, they are the second most studied macromolecular group. There are some data which demonstrate that high adsorption capacities can be achieved, although the numbers presented are not as high as for proteins. Finally, lipids are a large molecular group composed of a broad range of molecules differing significantly in structure and function. Fatty acids are mainly used as stabilizing agents on nanoparticles, reducing the colloidal size of the solids in suspension and creating a barrier to nanoparticle agglomeration. The studies of their interaction with nanoparticles concentrate predominantly on oleic and palmitic acids and their salts. Phospholipids such as DSPE or DMPE self-assemble onto naked IONPs as a coating. Both types of lipids are amphiphilic molecules that form bilayers. Few works in the literature focus on less amphiphilic (or completely hydrophobic) lipids. Some examples of very hydrophobic complex molecules, also associated with the group of the lipids, are, for instance, cholesterol and magnetoliposomes [139]. They also interact with IONPs for separation applications or as drug delivery system.

The studies cited in the sections above generally report individual molecule experiments, which provide insight into the adsorption mechanism for attachment to the IONPs surface. These studies pave the way to design and apply magnetic bioseparation methods to industrial processes, where the aim could be to purify a particular group of macromolecules. Studies of macromolecule families are, first, of special interest to fields such as the food and feed industries, where the whole proteome and lipidome of biotechnological mixtures is extremely valuable, and, second, to the energy industry, whose interest in developing novel bio-fuels suggests the need to obtain purified lipids and carbohydrates. This review gathers the essentials to enable the visualization, understanding and comparison of adsorption mechanisms of macromolecules to IONPs. However, macromolecules are found mixed in fermentation broths, where several elements take part in the competition for the nanoparticle surface, making individual macromolecules studies suboptimal and increasing the need for further competitive studies of several target molecule types from real cell lysates.

## From model systems to complex mixtures

The above-mentioned work includes thorough analyses made with model systems, where studies are carried out on specific molecules in controlled environments. In contrast, this section presents adsorption works based on biological mixtures.

### Physiological protein corona

When a nanoparticle enters into a physiological environment, proteins surround the surface instantly, giving it a biological identity and changing the synthetic identity. This is called the formation of the ‘protein corona’ [67]. Since 2007 the term ‘corona’ has been regularly used to denote the natural self-assembly of proteins onto the nanoparticle surface [53, 144].

Most of the published works, which are carried out with plasma or serum and aim at therapeutic usage, focus on competition among proteins [81, 145, 146]. When the IONPs enter the physiological media, the proteins which attach to the nanoparticle surface determine the fate and biocompatibility of the IONPs. Those studies offer insight into the identification of the adsorbed proteins, and elucidate the factors controlling nanoparticle-protein interactions. The entire plasma proteome is composed of 3700 proteins, of which only 50 have been reported to bind to various nanoparticles [52]. The binding is described with reference to the Vroman effect with the concentration of each protein as the determining factor for the initial adsorption behavior [147]. Over time, proteins of lower abundance but with higher affinity and slower exchange rates replace the first-attached proteins [147, 148]. However, the Vroman effect has been studied solely in mixtures of a few proteins, and these results likely do not correspond to the behavior in complex mixtures with other concentration profiles and broader biomolecular landscapes.

In the case of plasma proteins, albumins are replaced by fibrinogen and immunoglobulin G (IgG) and then substituted by IHRPs (inter-alpha-trypsin inhibitor family heavy chain-related proteins) and apolipoproteins [52]. Protein corona studies with nanoparticles other than iron oxides demonstrate, though, that this succession and the content of the hard corona can vary according with the concentration of plasma used [9], the hydrophobicity of the nanoparticle [52], the material and coatings applied [9, 149, 150], the composition of the protein source [151], or size and charge of the particles [92]. We also hypothesize that if the concentration of nanoparticles used is high in comparison to the protein concentration, there will be sufficient binding sites for all the proteins to co-exist in the corona without displacing low-affinity proteins.

Fetal bovine serum (FBS) protein corona has been investigated using different coated-IONPs, including PVA and dextran, finding that the negative and neutral surface charge of PVA-coated IONPs adsorbed more proteins than dextran-coated IONPs. The protein corona is comprised chiefly of serum albumin, serotranferrin, prothrombin, alpha-fetoprotein, and kininogen-1 [152].

Regarding the characteristics of the nanoparticle, Ashby et al. (2014) show the impact of modification of the hydrophobicity on IONPs surface [54]. The more hydrophobic the surface was, the more adsorbed hydrophobic protein was obtained, revealing a more dynamic protein corona comprised of proteins with high exchange rates. Hence, the modification of the surface properties, including coatings, strongly defines the composition of the protein corona [152–154].

One essential factor influencing the behavior of the protein corona is the medium, which affects the exchange of adsorbed and free proteins; it probably exerts a greater influence on the composition of the soft corona, which is also more sensitive to environmental changes. In contrast, the hard corona resists these changes and contains the proteins with slower exchange kinetics and thus, longer residence time on the particle [54].

Due to the fact that only physiological scenarios have been examined, the knowledge in this area is limited. Even though some studies characterize the types of proteins surrounding the nanoparticle surface [155], the heterogeneity in the analytical methods and the experimental parameters and conditions have led to gaps and conflicting conclusions. Thus, current and future research should take into account guidelines for carrying out and reporting on experimental work, such as those proposed by Chetwynd et al. (2019) and Faria et al. (2018), on bio-nano experiments, as a tool to enhance reproducibility, extend data mining for systematic comparisons and to ease in-silico and meta-analysis [156, 157].

### Biological corona

In complex environments, such as fermentation broths or biological complex media, the biomolecules compete with metabolites, buffer species and cell debris for the binding sites on the nanoparticle surface during adsorption. This adsorption is a dynamic process where the biocorona composition changes over time due to the particular association and dissociation rates of all species [158]. These rates define the affinity of the biomolecule to the nanomaterial and ensure successful adsorption at the equilibrium state [159].

**Fig. 3:**
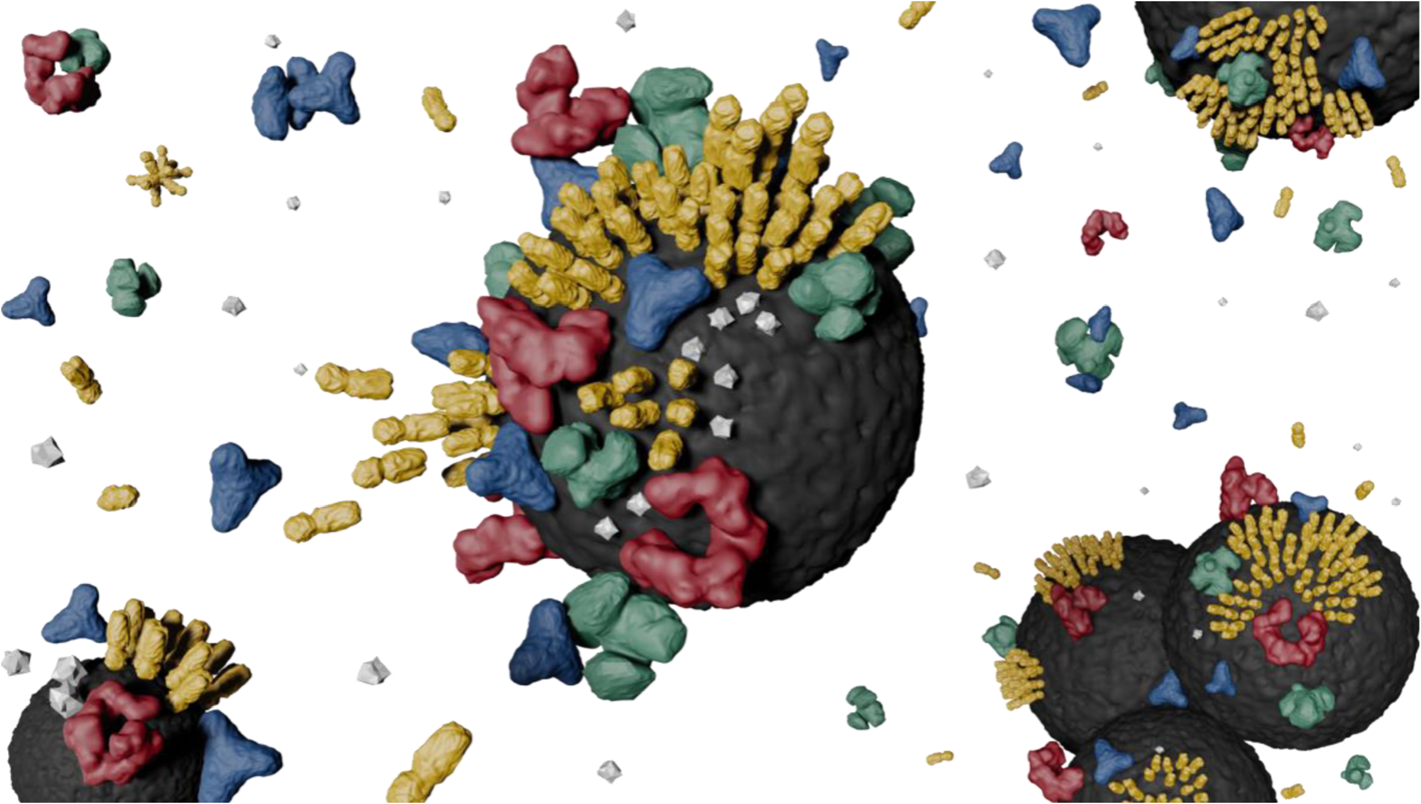
Illustration of biocorona formation. These molecules, however, are not all at the same scale, so they are not proportional. Proteins (represented in green), lipids (yellow), carbohydrates (blue), DNA (red), small molecules (grey) and ions form the biocorona in a biological milieu, where different phenomena can co-occur: (i) biomolecule-surface interactions, (ii) biomolecule-biomolecule interactions, for the formation of a mono-, bi- or multi-layer, and (iii) nanoparticle-nanoparticle interactions

Several studies include the broader concept of the biocorona or macromolecular corona [56, 160, 161], which remains insufficient however to describe the variety of elements involved in the adsorption process (as observed in Fig. 3), as inorganic ions and cell debris are also part of this composition. Additionally, despite the fact that the term biocorona is used, these studies again generally characterize protein behavior, relegating other biomolecules to a second plane. The other three large biomolecular groups have a wide range of molecular compositions, molecular weights, morphologies in solution and branching degrees, which makes their precise identification and quantification in real cell broths difficult [50].

Furthermore, it is important for the future to investigate symbiotic effects among different molecular classes. For instance, cholesterol and phospholipids, as part of the external area of lipoproteins, can bind onto copolymer nanoparticles of N-isopropylacrylamide (NIPAM) and N-t-butylacrylamide (BAM) [162]. The amount of binding depends on the hydrophobicity, leading to higher concentrations in more hydrophobic nanoparticles, i.e. 50:50 NIPAM:BAM, and on the specific surface area, obtaining greater amounts of lipids in the smallest nanoparticles (120 nm vs 200 nm). Additionally, a coupled binding behavior of lipids and lipoproteins was observed to be determinant in the saturation of nanoparticles´ surfaces [162]. This example suggests that other nanoparticles may also behave similarly. The possibility of such synergies has not been explored at all for iron oxide nanoparticles. Moreover, further surface analyses are necessary to better understand the binding mechanisms, as well as to more thoroughly investigate the existence of steric effects.

Several research projects have been conducted on the adsorption of polysaccharides from mixtures. Organic Matters (OM) are biological mixtures commonly found in natural waters, with a content that varies widely in concentration depending on the source. According to Vindedahl et al. (2016), the main components of OM are humic and fulvic acids, whose interacting mechanisms with iron oxides are diverse, but chiefly related to carboxylic and hydroxylic group interactions at the bio-nano interface and to charge differences between elements, primarily dictated by the environmental pH and surface protonation [163]. The quantity of humic acid adsorbed is highly dependent on the pH. In current works, the competition between polysaccharides and other macromolecules in extracellular polymeric substances (EPS) from cyanobacteria on nanoparticle surfaces has also been observed [164]. Such a mixture is composed mainly of polysaccharides, but also contains proteins, DNA and lipids. Although EPS can be seen to interact with the IONPs, no individual characterization of the macromolecules or analytics focusing on the interactions are presented in the study [164].

The framework applied by Basu et al. (2013) provides insights for research on biomolecules in competition for the IONPs surface, focusing on the isolation of Gram-positive and Gram-negative bacterial genomic DNA from mixtures, such as milk, fruit juice and pond water [14]. Although the results show a DNA yield around 20 μg, protein contamination at a A_260_/A_280_ ratio of 1.8 has been reported [14], which means that proteins had adsorbed on the surface as well.

As previously mentioned, DNA can tightly adsorb to IONPs via the phosphate backbone, but most proteins do not contain these groups. However, in other materials, such as gold, DNA attaches via the four nucleobases by means of coordinated interactions. Consequently, DNA competes differently with proteins. Additionally, gold surfaces have a strong affinity for proteins and DNA. Wu et al. (2020) studied the displacement of bovine serum albumin (BSA) and DNA oligonucleotides in gold nanoparticles [165], but as far as we know, no similar studies with IONPs are to be found.

In addition to competing for the surface, biomolecules can also create a symbiotic adsorption when they interact with each other, as previously mentioned. The direct attachment of proteins to the surface alters their structure, affecting their biological activity. However, an alternate deposition of proteins with a negative charge (e.g. BSA), and the polysaccharide glycol chitosan, with a positive charge, on IONP surfaces does not alter the secondary structure of the protein, enabling multilayer adsorption of both types of molecules [166]. As proposed by Chetwynd and Lynch (2020), a biocorona coexists with the protein corona, and small molecules are intertwined or bound with proteins [167]. The biocorona may have the same adsorption behavior as the protein corona, i.e. the most abundant and affine biomolecules define the nature of the surface [167].

### Future perspectives: moving towards a comprehensive biocorona analysis

We have limited this review to the largest classes of biomolecules. Future work should include other very interesting lipids such as steroids and water insoluble vitamins, as well as hybrid molecules like glyco- or lipoproteins. Moreover, data should be collected on the binding to the nanoparticle surface of small ions, such as atomic ions or buffer ions, and small organic acids, in interaction and in competition with bigger macromolecules.

Given the variety of biological aqueous suspensions, extending the characterization of the corona to systems other than the physiological one is crucial. Furthermore, the corona examination has to be expanded from the current focus (the protein corona) to include the behavior of lipids, polysaccharides, nucleic acids and small molecules, which also play a role in the corona identity and have rarely been included in such studies. The symbiotic effects among different molecular classes, which reduce free energy and enhance collaborative binding, must be better understood. Special emphasis should be put on analyzing the role of salt ions and small molecules on the interface, which is usually not considered when analyzing interaction driving forces, but which is instrumental in building the electrochemical double layer at the interface. Of particular interest is expanding research to real biotechnological mixtures from bacteria, yeasts, microalgae, etc., with their individual distributions of biomolecules.

The scientific community has made a remarkable effort toward understanding and controlling nanoparticle-biomolecule complexes, employing simulation and experimental methods, as well as kinetic and thermodynamic analysis. Despite all this work, gaps still exist with regard to developing analytical methods to interpret interactions, competition in multi-compound solutions and elution conditions. Elution is another key concept. Interaction is often seen as ‘from solution to the surface’, so-called adsorption, but desorption (recovery) is also immensely important in the bioseparation of target molecules. A more in-depth analysis and understanding of the driving forces for desorption is as important as for adsorption.

One necessity for a faster and broader advance in revealing binding mechanisms at solid-liquid interfaces are analytical techniques with sufficient resolution in a wet system. Methods currently applied principally include chromatography, optical spectroscopy, and other spectroscopy forms, for example infrared and Raman, as well as calorimetry and microscopy [92]. Unfortunately, high resolution equipment for spectroscopic or scanning microscopic measurements is not only expensive, but also works only in high to ultrahigh vacuums, which represent a completely different reality to that experienced by molecules in a dense and complex liquid mixture.

Finally, even if the goal of scientific investigation should be considered valuable in and of itself as leading to understanding the fundamentals, humanity must exploit this knowledge for new developments for our society. IONPs have the exciting property of superparamagnetism and are therefore a valuable tool for developing new bioseparation processes. However, for processing, suitable devices as well as a thorough design of the process steps are absolutely necessary. Recent reviews summarize the state-of-the-art in magnetic separation for biotechnological applications and demonstrate its future opportunities [168–170].

Additionally, we would like to encourage new perspectives on the description of bio-nano interactions. Adsorption on nanoparticulate systems is often a very fast process. Furthermore, the thermodynamic equilibrium of the adsorption reaction is commonly described using the Langmuir isotherm or other closely related models, such as Freundlich, Sips or Temkin. Nonetheless, such isotherms in nanoparticulate systems are not always useful for calculating affinity constants because, in the low concentration range, equilibrium values in solution are often so low that they result in analytical errors. The bio-nano interaction can also be analyzed from the perspective of an extraction of the target from the liquid to the solid, where the partitioning of the species between the solid surface and the liquid phase depends heavily on all species and their concentrations, as well as on their solubilities and hydration states. This perspective makes it easier to understand that for nanoparticulate systems, the target molecule can be found mainly adsorbed onto the surface depending on the existing concentration gradients. In such cases, few target molecules remain in solution. Such a scenario raises questions about the meaning of affinity constants from adsorption isotherms for these kinds of nanosystems.

## Conclusions

This review gathers recent studies on the adsorption of the four main biomacromolecules onto iron oxide nanoparticles. Particular attention is given to mixtures, which are essential for the assessment and further application of nanoparticles as a bioseparation tool. Starting from individual studies on model molecules, proteins are the most investigated molecules in interaction with IONPs. However, more research is necessary in order to include factors that are relevant during the adsorption process, such as steric effects and distribution, and conformation of the protein on the surface. These factors have only been rarely analyzed. The literature about DNA highlights the role of the phosphate group to form hydrogen bonds with the iron oxide surface, while carbohydrates and lipids are commonly used as coatings, where a forced interaction is carried out, often by means of a chemical reaction. All these studies help to illustrate the behavior of individual, model biomacromolecules. In mixtures, most of the knowledge is based on proteins and is connected with the description of the so-called protein corona. The study of lipid coronas is interesting due to its potential in biomedical applications, as this group represents an important constituent of blood plasma (e.g. lipoproteins). However, a holistic understanding and description of the biological corona is in its infancy as the main challenges continue to be the complexity and variability of the environments: the biome is vast, thus hampering the analytics. A deeper understanding of the macromolecules’ adsorption to IONPs is crucial, not only to elucidate the fundamentals behind the bio-nano interface, but also to exploit these concepts in bioseparation and other areas. A greater effort in the study of complex systems would multiply applications in all fields of life sciences and beyond.

## Data Availability

Not applicable.

## Abbreviations

DSP: Downstream processing
IONPs: Iron oxide magnetic nanoparticles
IEP: Isoelectric point
IgG: Immunoglobulin G
IHRPs: Inter-alpha-trypsin inhibitor family heavy chain-related proteins
PBS: Phosphate buffered saline
OM: Organic matter
GFP: Green fluorescent protein
CMC: Critical micelle concentration
NIPAM: N-isopropylacrylamide
BAM: N-t-butylacrylamide
PEG: Polyethylene glycol
EPS: Extracellular polymeric substances
BSA: Bovine serum albumin
APTES: (3-Aminopropyl) triethoxysilane
DSPE: 1,2-Distearoyl-sn-glycero-3-phosphorylethanolamine
DMPE: 1,2-Dimyristoyl-sn-glycero-3-phosphoethanolamine
PDP: Pyridyldithiol propionate
DOTAP: 1,2-Dioleoyl-3-trimethylammonium-propane
